# Gelatin-Based Hydrogels: Potential Biomaterials for Remediation

**DOI:** 10.3390/polym15041026

**Published:** 2023-02-18

**Authors:** Robson Andreazza, Amaia Morales, Simone Pieniz, Jalel Labidi

**Affiliations:** 1 Chemical and Environmental Engineering Department, University of the Basque Country UPV/EHU, Plaza Europa 1, 20018 San Sebastian, Spain; 2 Center of Engineering, Federal University of Pelotas, Gomes Carneiro 1, Pelotas 96010-610, Brazil; 3 Nutrition Department, Federal University of Pelotas, Gomes Carneiro 1, Pelotas 96010-610, Brazil

**Keywords:** hydrogel, gelatin, sustainability, remediation

## Abstract

Hydrogels have become one of the potential polymers used with great performance for many issues and can be promoted as biomaterials with highly innovative characteristics and different uses. Gelatin is obtained from collagen, a co-product of the meat industry. Thus, converting wastes such as cartilage, bones, and skins into gelatin would give them added value. Furthermore, biodegradability, non-toxicity, and easy cross-linking with other substances can promote polymers with high performance and low cost for many applications, turning them into sustainable products with high acceptance in society. Gelatin-based hydrogels have been shown to be useful for different applications with important and innovative characteristics. For instance, these hydrogels have been used for biomedical applications such as bone reconstruction or drug delivery. Furthermore, they have also shown substantial performance and important characteristics for remediation for removing pollutants from water, watercourse, and effluents. After its uses, gelatin-based hydrogels can easily biodegrade and, thus, can be sustainably used in the environment. In this study, gelatin was shown to be a potential polymer for hydrogel synthesis with highly renewable and sustainable characteristics and multiple uses.

## 1. Introduction

Hydrogels are characterized as three-dimensional cross-linked polymeric networks that can be produced with the most variable compounds and with the most variable uses [1]. In the last decade, hydrogels have been used in many applications and with high technical and economic viability which include biomedical and environmental areas.

Gelatin-based hydrogels are one kind of hydrogel in which gelatin is used as the cross-linked polymer and give the gel characteristics such as structure and texture. Many studies have promoted this kind of hydrogel for biomedical uses with promising and important characteristics such as tissue engineering [2,3] and/or drug delivery [4,5] with a high economic impact on society, medicine, and environmental purposes with high applicability.

The market for gelatin in 2020 rose to about USD 3.18 billion and is further expected to reach USD 4.08 billion by 2024 as forecasted in the same report [6]. The market of gelatin in 2013 was the highest in the food and beverages sector (28%), followed by nutraceuticals (25.8%) and pharmaceuticals (21%), while their use in the cosmetic industry was only 5.5% [7]. Along with the advancements in drug delivery, it caused the development of new recipients as novel dosage forms to fulfill specific functions which directly or indirectly influence the extent and or rate of drug release. This enhances the development of new and modified recipient sources that continue to emerge for better drug delivery performance [8].

Several studies have shown the high potential of hydrogels as green and renewable materials, with highly efficient and promising uses [1,9]. Gelatin-based hydrogels have several advantages due to their biocompatibility, biodegradability, and nontoxic features [10]. Furthermore, gelatin is a natural protein-derived material, and it has been used for the synthesis of medical hydrogels because of its non-immunogenicity and capacity for enhancing cell adhesion apart from its excellent biocompatibility [11].

Environmental problems such as pollution and contamination of water and soil have increased over the years of industrialization, mining, and the use of natural resources. Bioremediation and remediation processes come to assuage the contamination and promote environmental sustainability. In this way, hydrogels have a high potential for remediation with a substantial capacity for the adsorption of a wide range of pollutants such as toxic metals [12,13,14,15], organic compounds [16,17], dyes [18,19,20], and others.

New materials are well accepted by scientists and society for whatever their purpose is; however, when these new materials have innovative characteristics, they are also sustainable and can easily biodegrade in the environment, these products can be accepted in many countries and societies. So, the aim of this overview is to discuss, characterize, and indicate gelatin as a renewable source for hydrogels with many innovative applications such as biomedicine and environmental applications such as remediation for decontamination of water, effluents, and soil.

## 2. Hydrogels

Hydrogels are three-dimensional (3D) gel-like materials with hydrophilic functional groups in a polymeric chain and are capable of holding a large volume of hydro-fluids compared to their mass [21,22]. They can also be defined as water-absorbing natural or synthetic polymeric substances that swell im water and retain a significant amount of water within the structure without dissolving [23]. Hydrogels attract water due to the polar functional groups on the skeleton of the macromolecule and inhibit dissolving due to cross-linking. Two types of cross-linking, chemical or physical, can exist in the macromolecular chain.

Generally, hydrogels swell until the thermodynamic force of swelling is compensated by the elastic and retroactive strength of the cross-links [21]. The volume of water taken up by the macromolecule varies according to the structure of the hydrogel and the environmental conditions such as the pH, the temperature, and the ionic strength of the aqueous solution to which the polymeric network is exposed [21,22].

According to the nature of their components, hydrogels can be classified as synthetic (organic or inorganic), biological, and hybrid hydrogels. The structure of the polymeric network is controlled by gelling chemistry, leading to hydrogels with custom properties for multiple uses. Depending on the charged pendant groups on the main chain, synthetic organic hydrogels can be classified into ionic, neutral, and electronic conductor polymers [24]. Common examples of neutral polymers are poly(ethylene oxide) (PEO), poly(hydroxyethyl methacrylate) (PHEMA), and poly(vinyl alcohol) (PVA), which permit the formation of hydrogels via several cross-linking methods [24,25]. Moreover, biological hydrogels are potential materials due to their capacity of imitating the physical, chemical, and biological characteristics of tissues [26]. These hydrogels can have biologically active moieties such as gelatin, collagen, and elastin that enhance cell growth, migration, and proliferation [26]. Among these active moieties, gelatin has been extensively employed. Gelatin is obtained via the hydrolysis of collagen, which is the main protein present in the extracellular matrix of most tissues and can also be degraded enzymatically due to the metalloproteinase-sensitive sequences on its matrix [26]. Thus, it can promote biodegradation since it is a natural organic product and can be one sustainable characteristic of gelatin-based hydrogels.

Hydrogels can be produced with the most variable compositions and gel characteristics such as texture and structure. Many studies on different polymers promote these materials as innovative, green, and renewable materials with high importance for many uses [1]. Furthermore, these kinds of materials have been studied for biomedical and environmental applications [3,15,27]. Hence, their wide applicability and the broad formulation possibility with different compositions and polymers can increase their performance and make their use technically and economically viable.

In the modern world, emissions of pollutants and contamination of the environment have been important problems for humanity to solve and reduce. Here, hydrogels can be important since they show great adsorption capacity for contaminants and they can also be produced with different materials and be modified.

Many matrices such as gelatin [28], poly(vinyl alcohol) (PVA) [1], acrylamide [29], alginate dialdehyde-gelatin (ADA-GEL) [3], dialdehyde carboxymethyl cellulose-dextrin, and gelatin [30] have been used for the synthesis of hydrogels. Additionally, many other components have been added for increasing their adsorbent capacities; these include lignin [1], montmorillonite [29], gelatin ionically modified by bacterial cellulose [4], and graphene oxide/lapndonite/gelatin hydrogels [31].

The bio-based hydrogels possessing ion exchange and/or chelating groups have attracted much attention thanks to their large water portion, 3D structural morphology, swelling property, biodegradability, and non-toxicity [32,33]. Various moieties (i.e., amine (–NH_2_), hydroxyl (–OH), carboxyl (–COOH), and thiol (–SH)) on the surface of hydrogels can provide active binding sites and the 3D network structure can provide diffusion pathway to exhibit superior adsorption capacities and kinetics for the water pollutants [33,34]. Numerous biomaterials have been used for the fabrication of hydrogel-based adsorbents for wastewater treatment applications [33] and for pollutant removals such as chitosan [35], starch [12], xylan [36], sugarcane bagasse cellulose [20,37], biochar [12], or ulvan from green macroalgae [38]. However, when using an individual biopolymer it is more difficult to achieve high adsorption capacity and selectivity towards a specific pollutant or multicomponent adsorption. Thus, the incorporation of other functional compounds is a common approach to achieving the aforementioned properties. Therefore, surface modification of existing biomaterial with oxygen (O)-, nitrogen (N)-, or thiol (S)-containing functional groups has received widespread attention in wastewater treatment applications [33,39].

## 3. Gelatin-Based Hydrogels

Traditional gelatin is produced from animal origin, and it has been made from bones, cartilage, tendons, ligaments, and skin of animals such as cattle, pigs, fish, or chickens. As aforementioned, gelatin is derived from the partial hydrolysis of collagen protein presented in the previous sources [40]. Thus, due to the process of obtaining it, it may also be referred to as hydrolyzed collagen, hydrolyzed gelatin, or collagen peptide after it has undergone hydrolysis. After this process, the final product is colorless and soluble.

Gelatin is one of the most used ingredients with non-toxic and biodegradable characteristics in food and non-food industries for many purposes: for promoting gelation, stabilizing, thickening, emulsifying, and film forming [40]. Biodegradability is today an important issue since the sustainability of each product needs to exist; otherwise, the product might have problems with its future use because sanitary treatment is expensive and delimitation of space is a large problem for cities and governments. Even after obtaining the gelatin or the desired product from it, it has been demonstrated to have biodegradable characteristics, and it can contribute to producing new materials with high biodegradability potential inside the human organism [41] and in the environment [42,43].

Gelatin is used for giving consistency and viscosity to the hydrogels and, thanks to its aforementioned biodegradable and renewable nature, it can be an important source of sustainable hydrogels. Nowadays, the use of gelatin for the synthesis of hydrogels for multiple purposes depends on its structure and texture characteristics, and the applications range from the biomedical or biotechnological areas [2,3,30,44] to the most variable water and waste treatments [15,16,27,36,45]. Thus, gelatin-based hydrogels are versatile materials, which makes them very interesting. Some useful aspects of gelatin-based hydrogels employed for all the applications together with some others such as alimentary or environmental ones are displayed in Table 1.

As shown in Table 1, the wide applicability of hydrogels has been studied. In the biomedical field, for instance, purposes such as tissue engineering [3,30], cell culture scaffolding for bacterial growth and further human uses [48], dental pulp regeneration [49], capsules for drug delivery [4], and the treatment of myocardial infarction [31] have been investigated. Gelatin hydrogels are usually permeable to nutrients and oxygen, which enhance the survival rate of cells and their biological functions [48]. So, hydrogels with different compositions combining gelatin with other polymers could be useful for biomedical applications.

**Figure 1:**
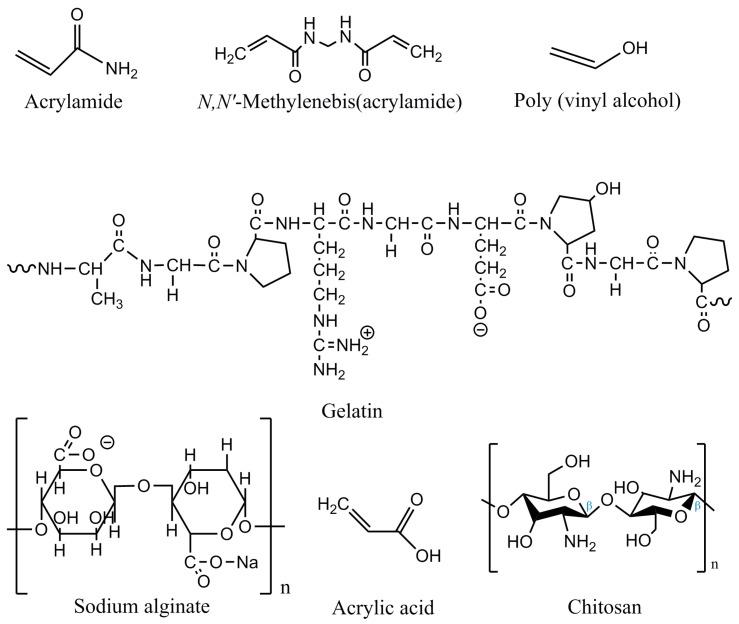
Chemical structure of some of the compounds that enable cross-linking with gelatin for the synthesis of gelatin-based hydrogels.

Gelatin is a protein that can easily biodegrade in the environment but has some stability limitations at high temperatures since once it has been dissolved, long durations at temperatures above 40 °C can promote protein denaturation. Moreover, gelatin is soluble in water, and this can also be a problem when synthesizing neat gelatin materials. As shown in Figure 1, gelatin is usually made from repeating units of glycine-X-Y. The high amount of the amino acids proline (12%), hydroxyproline (10%), and hydroxylysine (0.5%) make gelatin particularly special [50]. Depending on its origin, the content of proline and hydroxyproline might vary; in fact, fish gelatin is known for having a lower concentration of these amino acids compared to that coming from mammals. This affects negatively the gelling ability of fish gelatin, leading to a decrease in the gelation and melting temperatures together with a worsening of its mechanical properties, among others [51]. Thus, in order to synthesize more stable and resistant gelatin-based hydrogels, this biopolymer has been combined with many other polymers such as polyvinyl alcohol [28], alginate dialdehyde, alginate dialdehyde-gelatin reinforced with bioactive glass nanoparticles [3], dialdehyde carboxymethyl cellulose-dextrin [30], graphene oxide and laponite [31], collagen [49], chitosan [5], dopamine grafted, 1,4-phenylenebisboronic acid and graphene oxide [11], and oxidized alginate (OA) reinforced by silicon carbide nanoparticles (SiC NPs) and cross-linked with N-hydroxysuccinimide (NHS) and 1-ethyl-3-(3-dimethylaminopropyl) carbodiimide (EDC) [52]. Figure 1 shows the chemical structure of different compounds used for the synthesis of gelatin-based hydrogels.

### 3.1. Gelatin-Based Hydrogels Formulation and Preparation

Gelatin is a natural biopolymer with a high capacity for hydrogel formation with different compositions which promotes a proper cross-linking (Table 2). Green materials are really important for cleaning the environment since they have more acceptable characteristics for the sustainability of the environment. Thus, the composition of these materials is very important.

Depending on which kind of material, the concentration, composition, temperature, and time of reaction are mandatory for hydrogel formation. Additionally, if the hydrogel will be drying, the drying time and temperatures are variable (Table 2), and they are important too. Gelatin is a biodegradable protein with a temperature of denaturation starting above 40 °C [53]. So, it should be noted that all temperatures involved in the process of composition and preparation of the hydrogel should not be higher than 40 °C. Even if high temperatures may decrease the reaction time, when several components are involved in the reaction, it is still necessary to keep this temperature below 50 °C. In this way, high denaturation of the gelatin will be avoided, which is an important factor for the stability of the hydrogel. When different compounds with higher temperatures of solubilization such as PVA (around 90 °C) are used, it is important to dissolve these polymers before gelatin, then reduce the temperature, and after inserting gelatin in the formulation, avoiding premature degradation of the biopolymer. The physicochemical characteristics of the gel can influence its formation and stability and may be different for gelatins from the same source, according to the distribution of the amino acids and the extraction process used. The thermal stability of collagen is related to its content of amino acids (proline and hydroxyproline), and the higher the content of these amino acids, the greater stability of the triple helices. However, it must be highlighted that gelatin, when placed in cold water, can absorb 5 to 10 times its own mass and it swells. When this material is heated above its melting point (between 27 and 34 °C), the gelatin dissolves. This sol–gel transformation state is reversible. The thermal properties such as the melting and gelling point of gelatin are also related to the amount of proline and hydroxyproline amino acids in the original collagen [54]. Collagen denatures at temperatures above 40 °C generate a mixture of species with one, two, or three randomly coiled polypeptide chains [53]. Controlled cooling (below the melting temperature) leads to the recovery of a helical structure. Gels formed by gelatin can be considered as a partial return of molecules to an ordered state [55].

Gelatin can be the main cross-linking agent in the hydrogel; however, many other reagents can help with cross-linking, such as alginate [16,19], glutaraldehyde [19], chitosan [35,56], acrylic acid [57], *N*,*N*-methylene-bis-acrylamide [57], acrylamide [19], poly(vinyl alcohol) [58], and others. It is important to know the characteristics of the materials used in the composition of the hydrogels since they will determine the features of the synthesized hydrogel such as its stability, resistance, cross-linking, and reusability. Some materials used and listed above such as acrylamide or PVA can enhance the resistance and durability of hydrogels according to their concentration. These characteristics can determine their reusability in further adsorption processes, including the removal of other pollutants.

The reaction time is important for proper cross-linking. Many studies about hydrogels involving gelatin showed different reaction times, varying from several minutes to 24 h (Table 2). Actually, the reaction time will depend on their composition and can be modified with the different concentrations of the materials used. For instance, the cross-linking of gelatin hydrogels containing cassava flour (1%) and carbon-graphene oxide (1%) was performed in 5 min [20]. On the contrary, 24 h was needed in order to cross-link hydrogels with a more complex composition containing AM monomer (2.2%), HEMA monomer (0.2%), PVA (2.5%), cross-linking agent (EGDMA, 1.0% molar to total monomer), and TMEDA (5.0% molar to total monomer) [14]. 

In the molding and finishing of the hydrogels, it is necessary to remove the water and dry them via the most adequate technique and temperature (Table 2). Different forms such as oven-drying [15,37], room temperature [35], refrigerator, and lyophilizer [59] have been used. The time of drying can also be variable, ranging from 10 to 48 h, depending on the composition of the hydrogel and the method used for drying (Table 2). Temperature can also be varied, but high temperatures can initiate a partial denaturation as aforementioned, although some studies have shown drying temperatures up to 105 °C [20].

**Table 2:** Formulations of different gelatin-based hydrogels for remediation uses.

Hydrogel Formulation	Composition	Temp. of Mixture	Time of Reaction	Removal	Drying	Reference
Iron oxide	5% gelatin, 2 g FeCl_2_, 5.2 g FeCl_3_ (in 100 m)	70 °C	15 min	Yellow 12 (DY12)	60 °C	[18]
Cassava flour extracts and carbon-graphene oxide	1% cassava flour, 1% carbon-graphene oxide, gelatin (N.I)	NI *	5 min	Methylene blue trihydrate (MB) and direct red 23	48 h at 105 °C	[20]
Ba(OH)_2_ gelatin microcapsules	Gelatin (Ba/gelatin) different batches (0.16/1.5; 0.16/2.0; 0.24/2.0; 0.24/2.5)	80 °C	8 min	Sulfate	NI	[10]
Activated carbon- alginate -cyclodextrin	60% sodium alginate, 30% CD, 8% gelatin, 2% activated carbon	NI	30 min	2,4-dichlorophenol	24 h at 55 °C	[16]
Starch-gelatin mixed hydrogels with ferrite@biochar@molybdenum oxide	MoO_3_, CoFe_2_O_4_, starch, gelatin, glutaraldehyde. Concentrations NI	Microwave	NI	Pb (II)	50 °C	[12]
Chitosan	3% chitosan, 6% gelatin	50 °C	2 h	Acid orange II dye	30 °C	[56]
Chitosan—graphene bead	3% chitosan/3.2% gelatin/0.1–0.2% graphene	45 °C	Overnight	Orange II	Room temp.	[35]
Bentonite—bis-acrylamide—formaldehyde	Gelatin (20–40%), bentonite (1–3%), bis-acrylamide (200–500 μL), formaldehyde (50–200 μL)	NI	NI	Pb (II)	60 °C	[13]
Chitosan—acrylic acid—gelatin—*N*,*N*-methylenebisacrylamide	Chitosan (1%), acrylic acid (2.3%), gelatin (2–20%), *N*,*N*-methylenebisacrylamide (2.2%)	70 °C	3 h	Cu^2+^	60 °C	[57]
P(HEMA-co-AM)—PVA	AM monomer (2.2%), HEMA monomer (0.2%), PVA (2.5%), cross-linker (EGDMA, 1.0% molar to total monomer), TMEDA (5.0% molar to total monomer)	50 °C	24 h	Pb (II)	50 °C overnight	[14]
Sugarcane bagasse cellulose	0.5% gelatin, bagasse (NI)	40 °C	1 h	Cu (II)	50 °C in oven for 10 h	[37]
Alginate—acrylamide—ZnS nanocomposite	Sodium alginate and gelatin, acrylamide, glutaraldehyde (conc. varied)	60 °C	4 h	Biebrich scarlet and crystal violet dyes	60 °C air oven	[19]
Calcium—sodium bentonite	Calcium and sodium bentonite, gelatin (5%)	30 °C	NI	Clay turbidity, clay dosages, DBO	NI	[17]
Poly (vinyl alcohol) (PVA)	Poly (vinyl alcohol) (PVA)/gelatin, alginate (10% total mixture)	NI	2 h	Pb (II)	NI	[58]

* NI: not informed or not available in the published manuscript.

Some studies have demonstrated that natural gelatin-based hydrogels are usually soft and fragile, and hydrogels with elastic structures normally require modification of gelatin chains or amendment with chemical cross-linking agents [10]. These agents are needed to improve the structural characteristics of the hydrogels, giving them durability and/or stability.

### 3.2. Gelatin-Based Hydrogels for Remediation

Gelatin-based hydrogels for the adsorption of contaminants have many interesting advantages such as adaptable shapes, varying concentrations of the matrix polymer, adding charges with other materials, porosity, and permeability, for instance. Table 3 shows the variable compositions that gelatin-based hydrogels can have, containing different polymeric formulations for gel formation such as acrylamide, polyacrylamide, acrylic acid, *N*,*N*-methylenebisacrylamide and poly(vinyl alcohol) (PVA).

Many of the reported formulations for these hydrogels were used for the remediation of many organic and inorganic pollutants (Table 2). Some of the removed organic contaminants were direct yellow 12 [18], 2,4-dichlorophenol [16], acid orange II dye [56], biebrich scarlet, crystal violet [19], and methyl violet [45]. The potential of removal was high, achieving in some cases almost 99% of efficiency for the remediation of these organic compounds, most of them in aqueous solution but with potential use on water environments or effluents in waste treatment. 

The adsorption of methylene blue has also been studied for water decontamination. This contaminant decreases the water and environment quality. Some studies with gelatin-based hydrogels showed high efficiency for its remediation, and high concentrations of methylene blue were removed from an aqueous solution such as 26.04 mg of methylene blue g^−1^ of hydrogel composed of ethylene glycol, diglycidyl, xylan, and gelatin [36].

The incorporation of other materials such as colloids with high charge change capacity has many advantages for adsorption. Some authors demonstrated that adding montmorillonite into their hydrogels based on *N*,*N’*-methylenebisacrylamide improved the charges and also the adsorption of the toluene from an aqueous solution [29]. However, in the case of gelatin-based hydrogels, other mineral clays such as bentonite have been added for a high removal efficiency of Pb (II), which was higher than 90% and 47.169 mg for Pb (II) g^−1^ of the hydrogel [13]. Additionally, iron oxide can be important for adsorption since it increases the resistance and recalcitrance of the hydrogel. Another study showed that the incorporation of iron oxide for the removal of 197.4 mg L^−1^ of direct yellow from wastewater showed a high capacity of the hydrogel to remove this organic contaminant [18].

Cellulose and cellulose derivatives have useful chemical characteristics for tailoring hydrogels with wide applicability and high efficiency [9]. The incorporation of these materials into gelatin-based hydrogels has also been studied for environmental purposes. Marciano et al. [27] incorporated cellulose extracted from wastes into a gelatin-based hydrogel and achieved an adsorption of 13 mg of Cr (VI) per g of hydrogel, which could be used in water and waste treatment from different industry effluents.

Apart from the aforementioned inorganic heavy metal ions (Pb (II) and Cr (VI)), gelatin-based hydrogels have also shown to be promising for the removal of other heavy metals such as Cu (II), Co (II), Ni (II), and Zn (II) from wastewater and or water (Table 3). Some of these studies showed high concentrations for Cu (II) removal from water with 372.5 mg g^−1^ of Cu (II) using a hydrogel containing TiO_2_ nanoparticles and branched polyethyleneimine [59], and 261.08 mg g^−1^ of Cu (II) using a gelatin hydrogel containing chitosan, acrylic acid, and *N*,*N*-methylene-bis-acrylamide [57]. Thus, gelatin-based hydrogels also show a high potential for copper remediation.

Lead is a potential heavy metal with highly toxic characteristics, and this problem increases when it is bioavailable in water by solubilization. However, gelatin-based hydrogels can remove high concentrations of this heavy metal. In fact, more than 210 mg of removed Pb (II) g^−1^ hydrogel have been reported for gelatin/poly(vinyl alcohol) (PVA)-based hydrogels [58].

**Table 3:** Different compositions and uses of gelatin-based hydrogels for remediation.

Gelatin-Based Hydrogel	Pollutant	Situation of Application	Potential of Removal	Reference
Fe_3_O_4_	Direct yellow 12 (DY12)	Wastewater	197.4 mg L^−1^ of DY12	[18]
Sodium alginate, cyclodextrin, and activated carbon	2,4-dichlorophenol (2,4-DCP)	Wastewater and contaminated water	36.48 mg g^−1^ and 39.36 mg g^−1^ of 2,4-DCP	[16]
Chitosan and genipin	Acid orange II dye	Wastewater	0.25 mmol L^−1^ of acid orange II dye	[56]
Chitosan filled with graphene bead	Orange II	Wastewater	42.15 mg L^−1^ of orange II	[35]
ZnS, sodium alginate, and polyacrylamide	Biebrich scarlet and crystal violet	Water and wastewater	9.7 mg L^−1^ of biebrich scarlet and 28.63 mg L^−1^ of crystal violet	[19]
Grafted methyl methacrylate	Methylviolet (MV) dye	Water purification	49.5 mg L^−1^ of methyl violet (MV) dye	[45]
Ethylene glycol diglycidyl and xylan	Methylene blue	Water and wastewater	26.04 mg g^−1^ of methylene blue	[36]
TiO_2_ and PEI (GTP) aerogel	Oil/water separation Properties for both oil/water-free mixtures and oil-water emulsions and Cu (II)	Wastewater and water contaminated with oil spills	372.5 mg g^−1^ of Cu (II); 99.72% of oil rejection coefficient	[59]
*Ulva fenestrata* and dialdehyde	Methylene blue, Cu (II), Co (II), Ni (II), Zn (II)	Aqueous solution	465 mg g^−1^ of methylene blue; 14 mg g^−1^ for Cu (II), 7 mg g^−1^ for Co (II), and 6 mg g^- 1^ for Ni (II) and Zn (II)	[38]
Chitosan, acrylic acid, and *N*,*N*-methylene-bis-acrylamide	Cu (II)	Wastewater	261.08 mg g^−1^ of Cu (II)	[57]
Acrylamide, acrylic acid, *N*,*N’*-methylene-bis-acrylamide, sugarcane bagasse	Cu (II)	Water and wastewater	97.38 mg L^−1^ of Cu (II)	[37]
Bentonite	Pb (II)	Wastewater	47.169 mg g^−1^ of Pb (II)	[13]
Poly(vinyl alcohol) (PVA)	Pb (II)	Water and wastewater	210.43 mg g^−1^ of Pb (II)	[58]
Metal–organic frameworks UiO-66-NO_2_ film	Pb (II)	Water decontamination and food safety control fields	0.98 mg L^−1^ of Pb (II)	[15]
Cellulose incorporated from waste	Cr (VI)	Water and waste treatment	12 and 13 mg g^−1^ of Cr (VI)	[27]

## 4. Conclusions

Hydrogels have been described as promising materials over the years for many applications in diverse fields and areas. These materials have shown many interesting characteristics such as cross-linking, shape, and elasticity that can promote them as innovative materials with novel and important applications.

Gelatin-based hydrogels have been studied in the same way since gelatin is renewable, biodegradable, and can substitute expensive and oil-derived components for gel formation, leading to the production of green and non-toxic materials for humans and the environment. However, to improve the characteristics of gelatin-based hydrogels, and depending on their final application, it is important to combine gelatin with other sustainable materials such as waste of cellulose or lignin, or chitosan or mineral clays for improving their charge and structural characteristic. The key to the process of new polymers for hydrogels with gelatin is the use of other components.

To achieve a new polymer for a gelatin-based hydrogel, the concentration of the products, time of reaction, the temperature of the reaction, and temperature for drying the material are important to ensure no denaturation of the gelatin proteins and also the earlier degradation of the hydrogel. 

For environmental purposes, hydrogels have shown high performance as removers of many contaminants in aqueous solutions; however, as society is under continuous development, the type and amount of pollutants are constantly increasing and it is, therefore, still necessary to study the remediation of a wider variety of contaminants and mediums for increasing the spectrum of use of these materials in this field in the near future.

## Figures and Tables

**Table 1:** Different purposes, applicability areas, and potential uses of gelatin-based hydrogels.

Hydrogel	Purpose	Field	Potential Use	Reference
Poly(vinyl) alcohol-based	Mechanical and physical resistance	Biomedical	Economically high	[28]
Antioxidant peptides	Protective for enzyme	Biotechnology	Economically high for food industry	[44]
Gelatin nanoparticle	Tissue engineering, cell culture	Biotechnology	Economically high	[2]
Chitosan	Drug delivery	Biomedical	Economically	[5]
Dopamine grafted/ 1,4-phenylenebisboronic acid and graphene oxide	Tissue adhesives, wound dressings, and wearable devices	Biomedical	Economically high	[11]
Cellulose incorporation	Chromium adsorption	Environmental	Pollution control and waste treatment	[27]
Cellulose microcrystals incorporation	Drug delivery	Biomedical	Economically	[46]
Oxidized alginate	Cartilage tissue engineering	Biomedical	Economically and healthy high	[47]
Carrageenan and potassium sulfate	Foods, materials, and other fields	Multiple uses	Economically and industrially	[10]

## Data Availability

Data sharing is not applicable.
